# Chitin Nanofiber Elucidates the Elicitor Activity of Polymeric Chitin in Plants

**DOI:** 10.3389/fpls.2015.01098

**Published:** 2015-12-09

**Authors:** Mayumi Egusa, Hidenori Matsui, Takeshi Urakami, Sanami Okuda, Shinsuke Ifuku, Hirofumi Nakagami, Hironori Kaminaka

**Affiliations:** ^1^Department of Chemistry and Biotechnology, Graduate School of Engineering, Tottori UniversityTottori, Japan; ^2^RIKEN Center for Sustainable Resource ScienceYokohama, Japan; ^3^Graduate School of Environmental and Life Science, Okayama UniversityOkayama, Japan; ^4^Faculty of Agriculture, Tottori UniversityTottori, Japan

**Keywords:** chitin, nanofiber, elicitor, *Arabidopsis thaliana*, rice, *Alternaria brassicicola*, Pseudomonas syringae pv. *tomato* DC3000

## Abstract

Chitin, an *N*-acetyl-D-glucosamine polymer, is a component of fungal cell walls and a microbe/pathogen-associated molecular pattern that elicits plant defense responses. As polymeric chitin is difficult to handle due to its insolubility in water, many studies on chitin-induced immune responses have used water-soluble low-molecular weight chitin instead. Thus, it is unclear if polymeric chitin can induce resistance. Here, we examined the elicitor activity of chitin nanofiber (CNF) of submicron thickness prepared from polymeric chitin. CNF showed a high dispersing ability in water and induced both reactive oxygen species (ROS) production and chitin-induced defense-related gene expression in *Arabidopsis thaliana* seedlings. The *Arabidopsis chitin elicitor receptor kinase 1* (*Atcerk1*) mutant, which is impaired in chitin perception, also failed to respond to CNF. CNF exposure triggered ROS generation in suspension-cultured cells from *Oryza sativa*. Furthermore, pre-treatment of *Arabidopsis* leaves with CNF effectively reduced pathogen infection by both the fungus *Alternaria brassicicola* and the bacterium *Pseudomonas syringae* pv. *tomato* DC3000. These results demonstrate that CNF has elicitor activity and will help define the role of polymeric chitin in plant immune responses.

## Introduction

Chitin, an *N*-acetyl-D-glucosamine polymer, is a component of fungal cell walls and arthropod exoskeletons. As a biocompatible and biodegradable eco-friendly biopolymer, chitin has several promising applications in various fields. Due to its high nitrogen content and low C/N ratio, chitin can be used as a fertilizer or soil amendment to enhance crop growth. Moreover, chitin is expected to improve crop yields due to its ability to induce plant defense systems against pests and pathogens (Sharp, 2013).

Plant defense systems are activated in response to chitin in fungal (potential pathogen) cell walls, which is perceived as a microbe- or pathogen-associated molecular pattern (MAMP/PAMP). Bacterial flagellin, elongation factor Tu, lipopolysaccharides, and peptidoglycan are other examples of PAMPs, which are often highly conserved, constitutively expressed, and essential components of microbes (Antolin-Llovera et al., 2012). The recognition of PAMPs by pattern recognition receptors (PRRs) present at the plant cell surface induces PAMP-triggered immunity (PTI) (Antolin-Llovera et al., 2012).

The first plant PRR for chitin was identified in *Oryza sativa* (rice). Chitin elicitor binding protein (OsCEBiP) is a receptor-like protein (RLP) that contains an extracellular chitin-binding lysin motif (LysM) but lacks a known intracellular signaling domain (Kaku et al., 2006). OsCEBiP forms a complex with chitin elicitor receptor kinase1 (OsCERK1), a receptor-like kinase (RLK) that contains an active intracellular kinase domain, to initiate chitin signaling (Shimizu et al., 2010; Shinya et al., 2012). In *Arabidopsis*, AtCERK1 (also known as RLK1/LYK1) is essential for chitin signaling (Miya et al., 2007; Wan et al., 2008). AtCERK1 is an RLK that contains extracellular LysMs as well as an intracellular kinase domain. Chitin induces dimerization of AtCERK1 and activates immune responses, such as the generation of reactive oxygen species (ROS), activation of mitogen-activated protein kinases, and expression of defense-related genes (Miya et al., 2007; Wan et al., 2008). The biological activity of chitin elicitor depends on their size, with chitin heptamers to octamers showing high PAMP activity (Liu et al., 2012).

While several studies have examined chitin-induced PTI systems, chitins have not been widely used in practical applications. Because polymeric chitin is not soluble in most organic and inorganic solvents due to its high crystallinity (Pillai et al., 2009), many studies of chitin-induced PTI are based on water-soluble low-molecular weight chitin-oligosaccharides. Chitin only becomes soluble in water once it is costly degraded or chemically modified. Despite its huge availability, theses handling difficulties of polymeric chitin is a major obstacle for the utilization.

We recently developed chitin nanofiber (CNF) from polymeric chitin extracted from crab shell and mushrooms (Ifuku et al., 2009, 2011, 2012). Exoskeletons of crustaceans consist of CNFs. Chitin nanofibrils (∼3 nm in diameter) are embedded in a protein matrix and assemble into fibers (∼60 nm in diameter), and further these fibers assemble into micro-size bundles (Chen et al., 2008). Similarly, fungal cell walls consist of CNFs, which form a complex with glucans (Zivanovic et al., 2003). Extracted CNF has a highly uniform structure of 10–20 nm thickness and shows high dispersing ability in water due to its submicron size and high surface-to-volume ratio (Ifuku et al., 2011, 2012). In this study, we demonstrated that the polymeric CNF has elicitor activity in plants. We found that CNF induced ROS production and expression of defense genes and reduced pathogen infection in *Arabidopsis* and rice, similarly to chitin-oligosaccharide elicitors. We show that nanofibrillated chitin has useful applications for plant disease control.

## Materials and Methods

### Preparation of Chitin Nanofibers

Chitin powder from crab shell was purchased from Koyo Chemical (Tottori, Japan). CNFs were prepared without acetic acid as described previously (Ifuku et al., 2012). Briefly, dry chitin powder was dispersed in water at 1 wt.% and passed through a high pressure water-jet system (Star Burst Mini, HJP-25001S, Sugino Machine, Toyama, Japan) equipped with a ball-collision chamber for mechanical disintegration. Chitin-oligosaccharides (GlcNAc)_2-6_ and purified *N*-acetylchitohexaose (GlcNAc)_6_ were purchased from Yaizu Suisankagaku industry (Shizuoka, Japan).

### Plant Materials

*Arabidopsis thaliana*, ecotype Columbia (Col-8) and *cerk1-2* (GABI_096F09) were used. For inoculation tests, *Arabidopsis* plants were grown on sterilized soil [1:1 mixture of Supremix A (Sakata Seed Co., Yokohama, Japan), vermiculite] under controlled environmental conditions with 8 h light/16 h dark cycles at 22°C. For ROS assays and qRT-PCR, *Arabidopsis* seedlings were grown in liquid MGRL medium with 0.1% sucrose (Albert et al., 2006) at 22°C under continuous light for 10 days. Suspension-cultured rice cells derived from seed scutella of *Oryza sativa japonica* ‘Nipponbare’ were used. The rice cells were maintained using liquid L medium (Kuchitsu et al., 1993) on a rotary shaker at 25°C under dark conditions as described previously (Nakagami et al., 2010).

### Oligomeric Chitin Analysis

Oligomeric chitin in CNF was detected by HPLC analysis as described by Sashiwa et al. (2003). The water-soluble fraction from a suspension of chitin powder in water (10 mg/mL) and the filtrate from a CNF dispersant (10 mg/mL) through a Millex-HA filter (Merk Millipore, Darmstadt, Germany) were analyzed. Chitin-oligosaccharides [(GlcNAc)_2-6_] (10 mg/mL) were dissolved in water and used as a positive control. HPLC analysis was performed using a Hitachi HPLC system (Hitachi, Tokyo, Japan) equipped with a L-7100 pump, L-7200 autosampler, and D-7400 UV detector and conducted on a Shodex Asahipak NH2P-50 column with CH_3_CN/H_2_O (7:3, v/v) with the following settings: injection, 0.1 mL sample/CH_3_CN (1:2, v/v); flow rate = 1.0 mL/min; and UV detection at 210 nm.

### Chitinase Assay

Enzymatic degradation of chitin was analyzed by chitinase assay with Schales’ method as described by Ferrari et al. (2014). Un-nanofibrillated and nanofibrillated chitin (1 mg/mL) were incubated with chitinase (1.2 U, Wako Pure Chemicals Industries Ltd., Osaka, Japan) in 50 mM KPi buffer (pH6.0) at 30°C. Reactions were centrifuged at 4°C and 100 μL supernatant was mixed with 200 μL Schales’ regent (0.5 M sodium carbonate, 0.5 g/L potassium ferricyanide). The samples were incubated at 100°C for 15 min under dark conditions, and absorbance was then measured at 420 nm.

### ROS Assay

Three 10-day-old *Arabidopsis* seedlings were incubated in liquid MGRL medium supplemented with 0.1% sucrose containing 100 μM L-012 (Wako, Japan) for 2 h at 22°C under darkness, and then transferred to liquid MGRL medium containing 0.1% sucrose and chitin-oligosaccharides or CNF. ROS production was determined by counting photons derived from L-012–mediated chemiluminescence using a TriStar LB942 microplate reader (Berthold technologies, Germany). Similarly, 40 mg rice cells was incubated with liquid L medium containing 1 mM L-012 for 2 h at 25°C under dark conditions, and then with liquid L medium containing chitin-oligosaccharides or CNF and horseradish peroxidase (final conc. 1 unit, Sigma–Aldrich, USA).

### RNA Isolation and qRT-PCR Analysis

*Arabidopsis* seedlings (10-day-olds) were treated with 0.1 mg/mL CNF or water. Samples were harvested 1 h after treatment and frozen immediately. Total RNA was isolated using the RNeasy Plant Mini Kit (Qiagen, Netherlands) and cDNA was prepared using the ReverTra Ace Reverse Transcription Kit (Toyobo, Japan). Quantitative real-time PCR (qRT-PCR) was performed using the Mx3000P QPCR system (Agilent Technologies, Santa Clara, CA, USA) with Thunderbird SYBR qPCR Mix (Toyobo, Japan). Data were analyzed using an in-house script written in the R language as described by Tsuda et al. (2013). The gene-specific primers used were as follows: *FRK1* (At2g19190) FW 5′-ACGGGCATAGTTCCACAAAG-3′, *FRK1* RV 5′-CGTCAAAAGAACGACGATGA-3′; *CYF81F* (At5g57220) FW 5′-AATGGAGAGAGCAACACAATG-3′, *CYF81F* RV 5′-ATACTGAGCATGAGCCCTTTG-3′; *WRKY22* (At4g01250) FW 5′-TCCTTCGGAGAGATTCGAGA-3′, *WRKY22* RV 5′-CTGCTGCTACATGGCACACT-3′; *ZAT10* (At1g27730) FW 5′-TGTCACGCAACTTCCTTCT-3′, *ZAT10* RV 5′-TGGTGTCACTTTATGCTTATTC-3′; lectin-like protein gene (At3g16530) FW 5′-ACAATGCAGATTCACAAACTC-3′, lectin-like protein gene RV 5′-GCAAACGATACCTAGCCAA-3′; *Actin-2* (At3g18780) FW 5′-GTTGGTGATGAAGCACAATCCAAG-3′, *Actin-2* RV 5′-CTGGAACAAGACTTCTGGGCATCT-3′.

### Pathogen Inoculation

*Arabidopsis* plants were sprayed with distilled water, 1 mg/mL chitin-oligosaccharides [(GlcNAc)_2-6_], or 0.1 or 1 mg/mL CNF (including 0.01% silwet L-77) 24 h before pathogen inoculation. *Alternaria brassicicola* isolate O-264 was maintained on potato dextrose agar medium. O-264 was incubated on V-8 juice agar for 2–3 days at 25°C in the dark and spores were obtained. Droplets (10 μL) of O-264 spore suspension (10^4^ spores/mL) were placed on the leaf surface. Inoculated plants were kept under high humidity conditions in a moist chamber with a 10-h photoperiod at 22°C and lesion formation was observed 4 days post inoculation. *Pseudomonas syringae* pv. *tomato* DC3000 (*Pst* DC3000) was grown on KB medium containing rifampicin (50 μg/mL). Prior to inoculation, bacteria were suspended in 10 mM MgCl_2_ to a density of OD_600_ = 0.0002. *Arabidopsis* leaves were syringe-infiltrated with bacterial suspension. Inoculated plants were incubated in a moist chamber under a 10-h photoperiod at 22°C. To determine the bacterial population, inoculated leaves were harvested and cut into 1-cm^2^ samples at 3 days post inoculation. Samples were homogenized in 10 mM MgCl_2_ and a series of diluted samples were plated on KB medium containing rifampicin and cycloheximide (50 μg/mL). The number of colonies per plate were counted.

## Results

### CNF Consists of Polymeric Chitin

**FIGURE 1:**
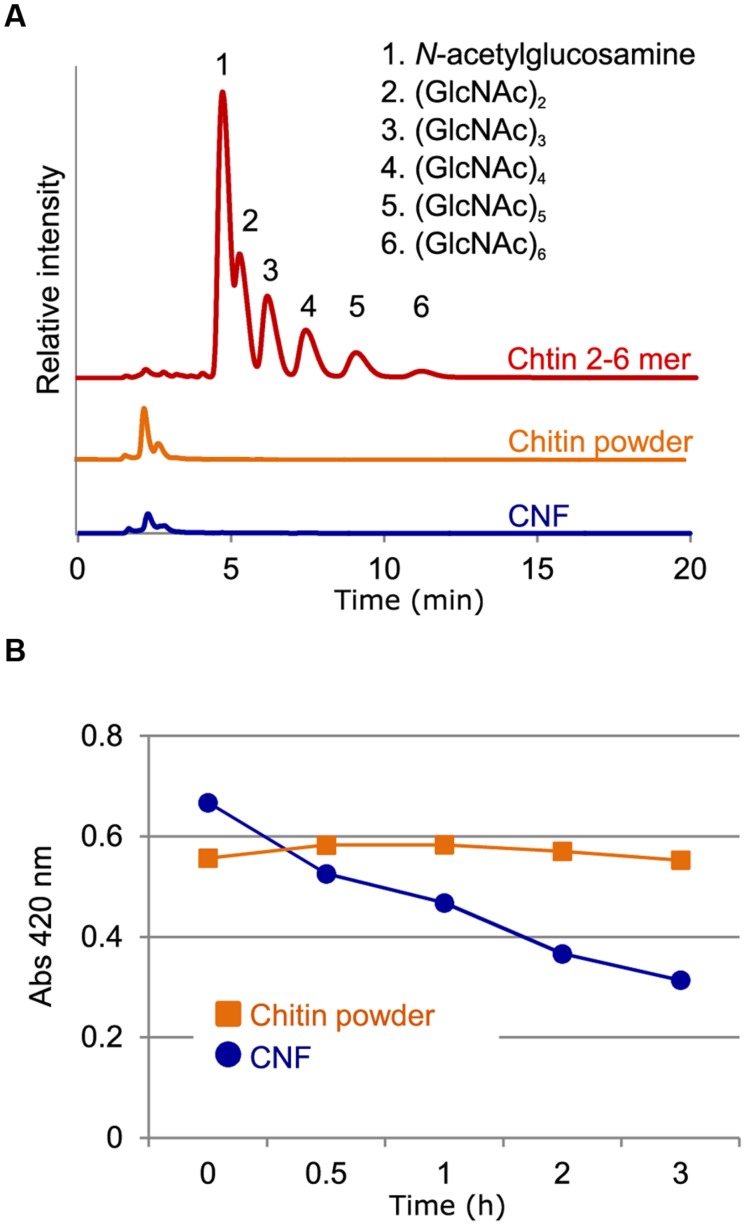
**Characterization of chitin nanofiber. (A)** Chitin nanofiber consists of long polymeric chitin. The water-soluble fraction from chitin powder suspension (chitin powder) and chitin nanofiber dispersant (CNF) were subjected to HPLC analysis. Chitin-oligosaccharides (GlcNAc)_2-6_ (Chitin 2-6 mer) were used as the positive control. **(B)** Comparison of chitinolytic enzyme sensitivity. Enzymatic degradation of chitin before (Chitin powder) and after nanofibrillation (CNF) was analyzed using a chitinase assay. Similar result obtained in four independent experiments and the data show one representative.

We previously described the preparation of CNF using the Star Burst system, which employs high-pressure water jet technology (Ifuku et al., 2012). In this process, chitin powder dispersed in water is passed through the Star Burst system under high pressure and atomized via collision with a ceramic ball. After these mechanical treatments, CNF of 10–20 nm thickness is obtained in slurry form and is highly dispersed in water (**Supplementary Figure S1**). We performed high-performance liquid chromatography (HPLC) to assess whether chitin fibrils were reduced in length as well as thickness during this mechanical process. There were no obvious peaks of chitooligosaccharides from the aqueous fraction of un-nanofibrillated chitin powder (**Figure 1A**). Moreover, filtrate from CNF did not contain oligomeric chitin (**Figure 1A**). These results confirm that chitin was not disintegrated in length during preparation and that CNF consisted of polymeric chitin.

### CNF is Rapidly Degraded by Chitinase

The unique properties of nanofibers come from their nanoscale size and high specific surface area, which provide greater access to the constituent molecules. We conducted chitinase assays to assess whether nanofibrillated chitin had increased sensitivity to chitinolytic enzymes. Whereas un-nanofibrillated chitin powder was not degraded over the course of 3 h, CNF was rapidly degraded (**Figure 1B**). These results suggest that in contrast to un-nanofibrillated chitin, which consists of chitin aggregates, CNF is composed of loosened chitin fibers that provide increased access to chitinase for degradation.

### CNF Induces ROS Production

**FIGURE 2:**
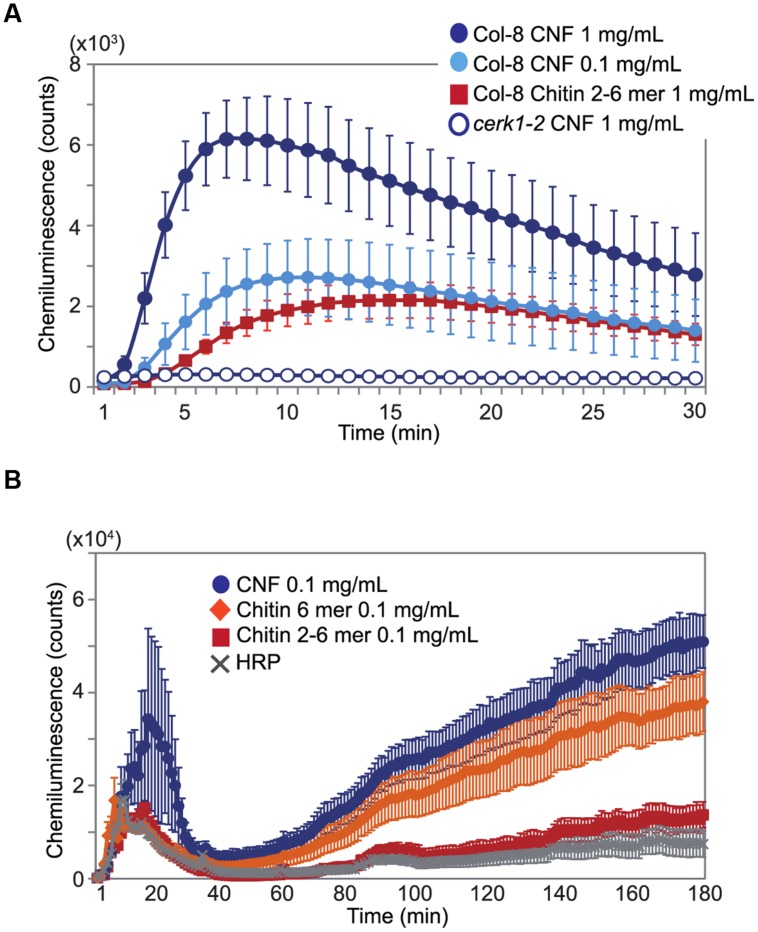
**Generation of reactive oxygen species (ROS) by chitin nanofiber treatment.** ROS production was measured by chemiluminescence mediated by L-012 after elicitor treatment. **(A)** ROS production in 10-day-old *Arabidopsis* seedlings. Wild-type Col-8 or *cerk1-2* mutant plants was treated with 1 mg/mL or 0.1 mg/mL chitin nanofiber (CNF) or 1 mg/mL chitin-oligosaccharides (GlcNAc)_2-6_ (Chitin 2–6 mer). **(B)** ROS production in suspension-cultured rice cells treated with 0.1 mg/mL CNF, chitin-oligosaccharides (GlcNAc)_2-6_ (Chitin 2–6 mer) or *N*-acetylchitohexaose (Chitin 6 mer). HRP: horseradish peroxidase. The values represent the average and standard errors of six replicate experiments.

Chitin elicitor is known to elevate ROS levels in *Arabidopsis* (Miya et al., 2007). As shown in **Figure 2A**, treatment with chitin-oligosaccharides [(GlcNAc)_2-6_] induced ROS production in *Arabidopsis* seedlings. We found that CNF was also capable of inducing ROS generation and that the induction was faster and higher than with chitin oligomers in wild-type Col-8. CNF-induced ROS generation was abolished in the *cerk1-2* mutant, which is impaired in chitin recognition (**Figure 2A**), indicating that CNF has elicitor activity mediated by the chitin receptor CERK1 in *Arabidopsis*. Maximal activation of innate immunity requires long-chain chitin oligomers (Liu et al., 2012). Whereas purified *N*-acetylchitohexaose (GlcNAc)_6_ induced the generation of more ROS than did CNF at the same concentration (**Supplementary Figure S2**), both CNF and (GlcNAc)_6_ induced ROS generation in a dose-dependent manner. Chitin-oligosaccharide elicitor induces biphasic generation of ROS in suspension-cultured rice cells (Yamaguchi et al., 2005). Here, we found that treatment of cultured rice cells with CNF induced biphasic ROS generation (**Figure 2B**). In contrast to our findings in *Arabidopsis*, ROS generation in cultured rice cells was higher for CNF than for (GlcNAc)_6_. These results indicate that polymeric CNF can be recognized by plants to trigger ROS production.

### CNF Induces Chitin-inducible Gene Expression

**FIGURE 3:**
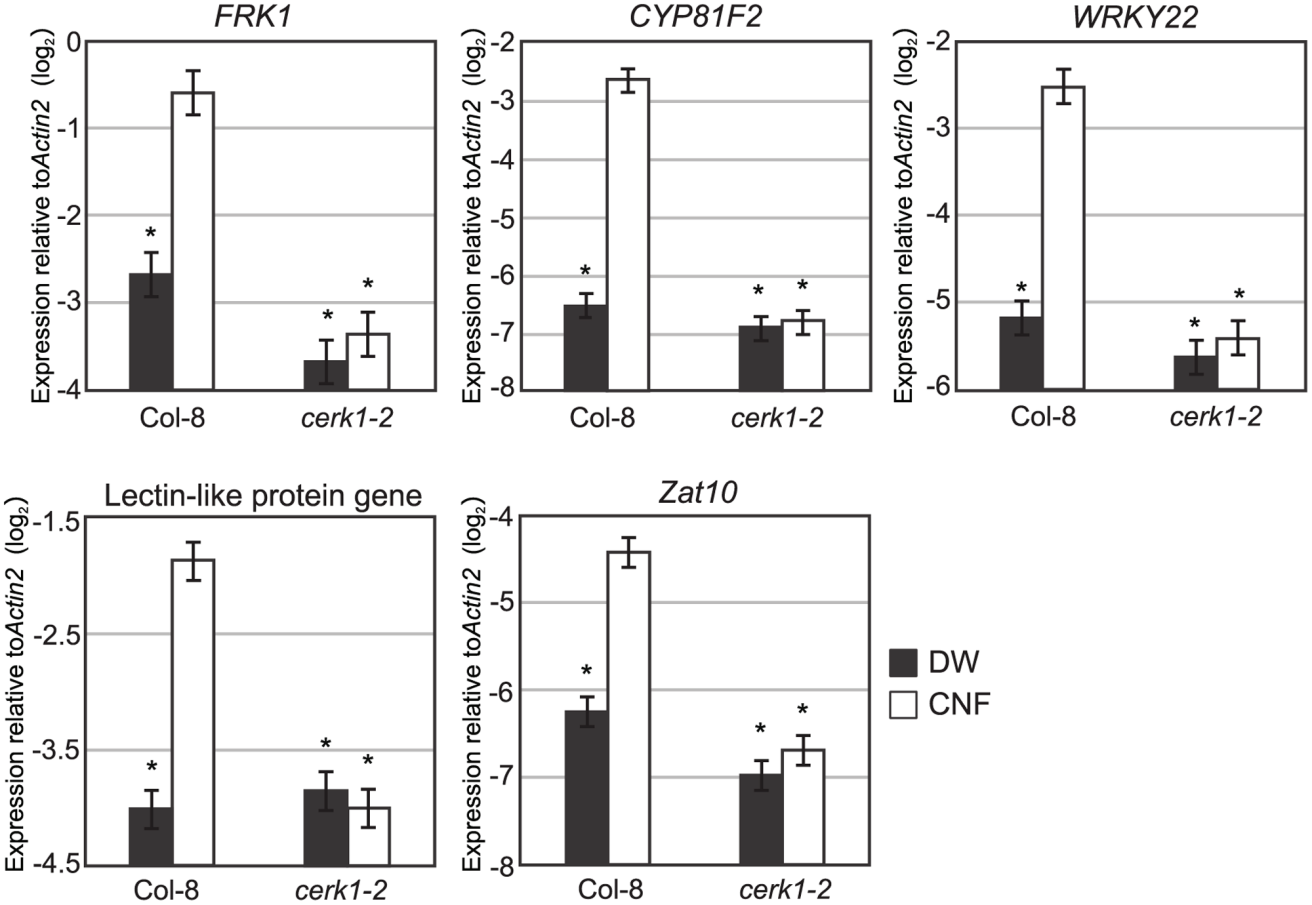
**Chitin-responsive genes in *Arabidopsis* respond to chitin nanofiber.** Ten-day-old seedlings of wild-type Col-8 or *cerk1-2* mutant were treated with water (DW) or 0.1 mg/mL chitin nanofiber (CNF). Expression of chitin-responsive genes was measured by qRT-PCR. Data represent means and standard errors of two biological replicates calculated by the mixed linear model. Asterisks indicate significant differences from CNF-treated Col-8 (*P* < 0.01, two-tailed *t*-tests).

Expression of defense-related genes such as lectin-like protein gene, *Zat10*, *WRKY22*, *FRK*, and *CYP81F2* are up-regulated by chitin-oligosaccharide treatment (Ramonell et al., 2002; Zhang et al., 2002). To investigate whether long-chain polymeric chitin induces the expression of defense-related genes, we examined the expression of these genes in *Arabidopsis* seedlings 1 h after CNF treatment by quantitative reverse transcriptase-PCR (qRT-PCR). As shown in **Figure 3**, all selected chitin-responsive genes were significantly up-regulated by CNF treatment in Col-8. By contrast, the expression of these genes was not responsive to CNF treatment in the *cerk1-2* mutant. These results indicate that CNF is an active elicitor capable of enhancing defense-related gene expression in a CERK1-dependent manner.

### CNF Reduces Fungal and Bacterial Disease Symptoms

**FIGURE 4:**
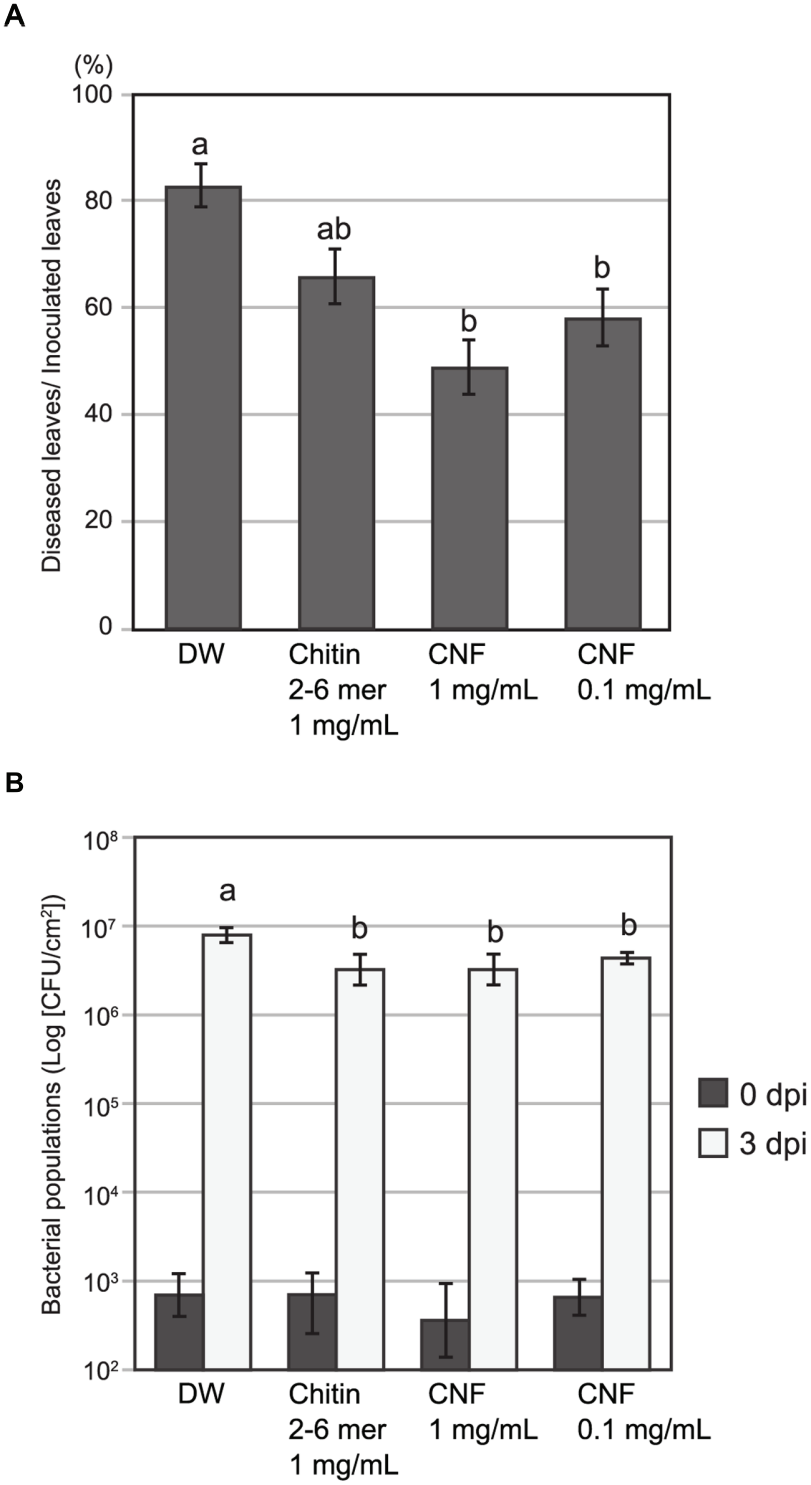
**Pre-treatment with chitin nanofiber reduces fungal and bacterial disease in *Arabidopsis*.** Plants were sprayed 24 h before pathogen inoculation with chitin nanofiber (CNF) (1 or 0.1 mg/mL), chitin-oligosaccharides (GlcNAc)_2-6_ (Chitin 2–6 mer) (1 mg/mL), or distilled water (DW). **(A)** Pre-treated *Arabidopsis* leaves were inoculated with fungal pathogen *A. brassicicola*. Lesion formation was observed 4 days after inoculation. Data indicate the rate of diseased leaves per all inoculated leaves and represent the mean and standard errors of six independent experiments. Means with the same letter are not significantly different according to Tukey’s test (*P* < 0.05). **(B)** Pre-treated *Arabidopsis* leaves were infiltrated with bacterial pathogen *Pseudomonas syringae* pv. *tomato* DC3000. Leaf disks were collected 0 and 3 days after inoculation to determine bacterial growth. Data represent the means and standard errors of three independent experiments. Means with the same letter at 3 dpi are not significantly different according to Tukey’s test (*P* < 0.1).

Chitin treatment induces resistance in host plants against both fungal and bacterial disease (Wan et al., 2008; Gimenez-Ibanez et al., 2009). To assess the effect of pre-treatment with long-chain polymeric CNF on pathogen infection, we inoculated *Arabidopsis* with the fungal pathogen *A. brassicicola* or bacterial pathogen *Pst* DC3000. Because of its high dispersing ability in water, we were able to apply CNF homogenously by spraying. Necrotic lesion formation upon *A. brassicicola* infection was reduced on leaves that were pre-treated with chitin-oligosaccharides [(GlcNAc)_2-6_] or CNF compared with control leaves (**Figure 4A**). Pre-treatment with 1 mg/mL or 0.1 mg/mL CNF significantly reduced lesion formation and the reduction was greater for the higher concentration of CNF.

To examine resistance to bacterial pathogens, we pre-treated *Arabidopsis* leaves with oligomeric chitin or polymeric CNF before infiltrating them with bacterial suspension. The population of *Pst* DC3000 at day 0 was not different and increased in all leaves 3 days post inoculation. However, the bacterial population in leaves pre-treated with chitin elicitors was slightly, but significantly, lower than that in the control leaves (**Figure 4B**). These results suggest that CNF is an effective elicitor for reducing infection of both fungal and bacterial pathogens.

## Discussion

Chitin elicitor triggers ROS generation, defense gene expression, ion flux, phytoalexin production, and disease resistance in both dicot and monocot plants (Shibuya and Minami, 2001). In this study, we showed that polymeric CNF is capable of inducing ROS generation and chitin-responsive gene expression (**Figures 2** and **3**) as well as resistance against pathogen infection in *Arabidopsis* (**Figure 4**). These results indicate that plants can recognize and respond to long chain polymeric chitin. Previous studies have reported that AtCERK1 binds to polymeric chitin and plays an essential role in chitin signaling in *Arabidopsis* (Petutsching et al., 2010; Wan et al., 2012). In our study, the ROS generation and gene expression responses to CNF were impaired in the *cerk1-2* mutant (**Figures 2** and **3**). These findings demonstrate that CNF induces PTI through CERK1, similarly to chitin-oligosaccharide. Although AtCERK1 was proposed to function both in chitin perception and signaling, despite its low chitin binding affinity (Liu et al., 2012; Cao et al., 2014), AtLYK5, which has a higher chitin binding affinity, was recently shown to be the primary chitin receptor (Cao et al., 2014). AtLYK5 is required for AtCERK1 dimerization and phosphorylation in a chitin-dependent manner (Cao et al., 2014). Polymeric chitin was bound to AtLYK5 *in vitro* (Petutsching et al., 2010), and it will be interesting to explore the use of CNF as a ligand in future research.

Chitin-oligosaccharide elicitor induces resistance to the fungi *A. brassicicola* and *Erysiphe cichorasearum* in *Arabidopsis* (Wan et al., 2008) and to *Magnaporthe grisea* in rice (Kouzai et al., 2014). Furthermore, chitin-oligosaccharide elicitor is also effective against bacterial pathogens (Wan et al., 2008); even though bacterial pathogens do not contain chitin, chitin elicitor signaling through AtCERK1 has an apparent effect on PTI against *Pst* DC3000 infection (Gimenez-Ibanez et al., 2009). We showed here that CNF efficiently reduced both *A. brassiciola* and *Pst* DC3000 infection (**Figure 4**). Despite the diversity of PAMPs and its corresponding PRRs, PTI events are largely overlapping (Antolin-Llovera et al., 2012). PTI is generally effective against non-specific and wide range of pathogens. Because public concern for environmental and biological systems is growing, ideal disease management should be safe for human and animals and eco-friendly. MAMPs are candidate substances for sustainable crop protection (Burketova et al., 2015). We show that nanofibrillated chitin could be practical material for plant disease control.

While the ability of chitin to induce resistance in plants is evident, chitin has not been widely used in agricultural applications. There have been a few attempts to use chitinous waste from edible mushrooms and crustaceans in agriculture for nutrition or soil amendment to enhance crop growth (Sharp, 2013). However, the application of chitinous compost in open fields had no discernable effect on disease control. Chitin must first be released from complex with protein or glucans in chitinous waste and nanofibrous structure or oligomeric fragment of chitin can be recognized by plants. Some reports have suggested that degradation of polymeric chitin to oligomeric chitin is required for recognition by PRRs (Stacey and Shibuya, 1997; Shibuya and Minami, 2001). As it would take time for chitin fragments to be released from compost, any effects of chitinous compost on disease resistance would likely be slow-acting. We demonstrated here that CNF was degraded by chitinase more rapidly than was un-nanofibrillated chitin (**Figure 1B**). Chitin fragments could be released and recognized by PRRs soon after CNF treatment; therefore, CNF may be a useful fast-acting elicitor. In addition, it was suggested that smaller fragments of chitin are not absolutely required for chitin recognition on account of strong binding of polymeric chitin to AtCERK1 (Petutsching et al., 2010; Cao et al., 2014). The timing of ROS generation induced by CNF was comparable with that induced by chitin-oligosaccharides (**Figure 2**), which suggests that polymeric CNF could be directly recognized by plant PRR. These findings indicate that the CNF nanostructure allows PRRs rapid access to polymeric CNF for initiation of PTI.

In summary, we have demonstrated that nanofibrillated polymeric chitin shows elicitor activity to induce ROS production and defense-related gene expression. Further, CNF effectively reduced the symptoms of both fungal and bacterial infection. Thus, using nanofibrillation to produce CNF of submicron size and high surface-to-volume ratio, and therefore much greater dispersibility in water, makes it possible to elucidate the elicitor activity of polymeric chitin. Our results also show that nanofibrillated chitin could be a useful and practical material for plant disease control in agriculture. Further study is needed to improve the material properties of CNF to enhance its elicitor activity for a broad range of host plants.

## Author Contributions

ME and HM wrote the main manuscript text. ME, HM, SI, HN, and HK conceived and designed the experiments. ME, HM, TU, and SO performed research. All authors reviewed and approved the manuscript.

## Conflict of Interest Statement

The authors declare that the research was conducted in the absence of any commercial or financial relationships that could be construed as a potential conflict of interest.
