# Supplementary figures and images for: Chitin Nanofiber Elucidates the Elicitor Activity of Polymeric Chitin in Plants
Source: Front Plant Sci. 2015 Dec 9;6:1098. doi: 10.3389/fpls.2015.01098 (PMC4673310; doi:10.3389/fpls.2015.01098)

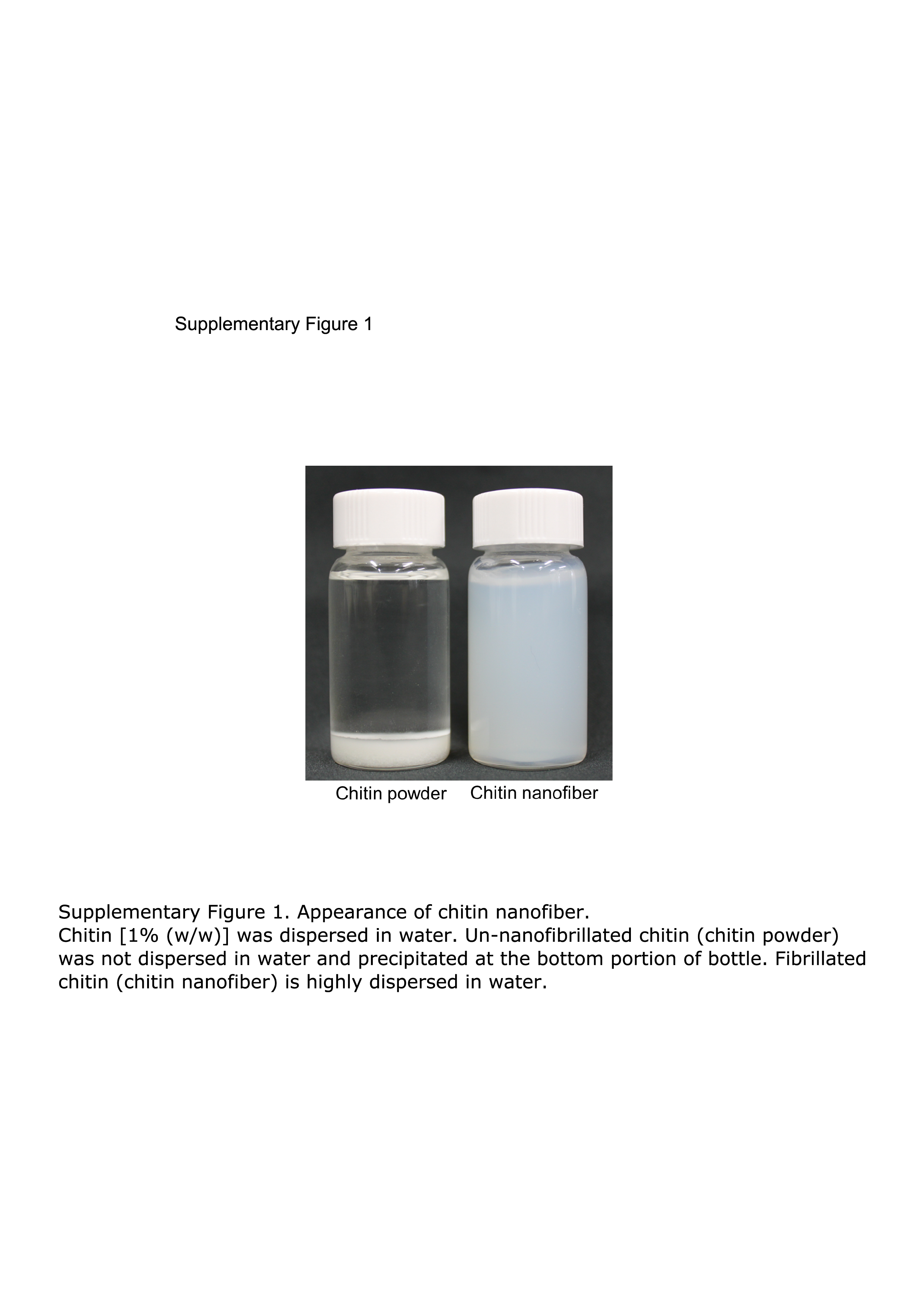

Supplement: Supplementary file 1 [file Image_1.TIF]

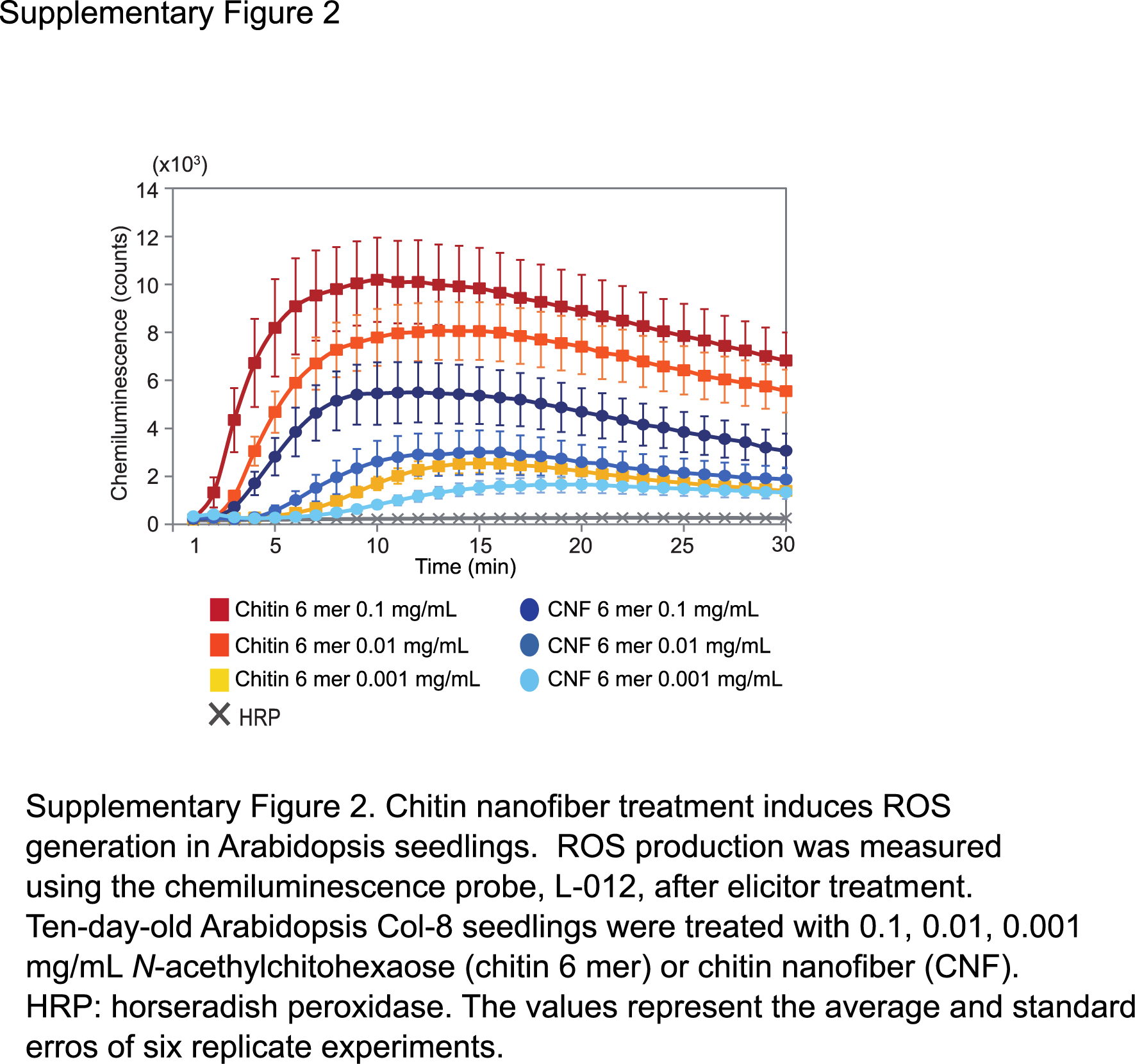

Supplement: Supplementary file 2 [file Image_2.TIF]
